# Rotavirus G5P[6] in Child with Diarrhea, Vietnam

**DOI:** 10.3201/eid1308.061038

**Published:** 2007-08

**Authors:** Kamruddin Ahmed, Dang Duc Anh, Osamu Nakagomi

**Affiliations:** * Nagasaki University Graduate School of Biomedical Sciences, Nagasaki, Japan; † National Institute of Hygiene and Epidemiology, Hanoi, Vietnam

**Keywords:** Human rotavirus, serotype G5, Vietnam, dispatch

## Abstract

We detected rotavirus G5P[6] with a long RNA pattern in a Vietnamese child with diarrhea. Viral outer capsid protein VP7 and VP4 genes suggest that it likely originated from porcine rotavirus either by genetic reassortment or as whole virions. To our knowledge, this is the first report of human rotavirus G5 in Asia.

Rotaviruses cause 352,000–592,000 deaths per year in children <5 years of age (*1*). More than 80% of these deaths occur in developing countries (*1*). In multicenter trials, monovalent and pentavalent rotavirus vaccines were safe and highly efficacious for children in developing countries (*2*). Among developing countries in Asia, Vietnam is progressing rapidly toward introducing rotavirus vaccine (*3*). Rotavirus accounts for 55% of diarrheal diseases in Vietnam, prevalent serotypes are G1, G2, G3, G4, and G9, and 3% of rotaviruses were untypeable (*3*). This low percentage of untypeable strains was unanticipated because it is assumed that developing countries are the source of unusual rotavirus strains (*4*). Many people in Vietnam live in close contact with domestic animals, which may promote interspecies transmission of rotaviruses and reassortment of human and animal rotaviruses. Therefore, we hypothesized that in rural Vietnam, some rotavirus infections are caused by animal rotaviruses or animal–human reassortants.

## The Study

We conducted a study by using double-stranded RNA obtained from 38 untypeable samples collected from children <5 years of age with rotavirus infections who were admitted to Khanh Hoa General Hospital and Ninh Hoa Hospital, Khanh Hoa Province, Vietnam. RNA was extracted from rotavirus-positive stool samples by using RNaid kits (MP Biomedicals/Qbiogene, Solon, OH, USA). Rotavirus-positive stool samples were detected by using Rotaclone ELISA kits (Meridian Bioscience. Inc., Cincinnati, OH, USA). Stool samples were collected from October 2003 through March 2004 as part of a rotavirus surveillance project conducted by 1 of the authors (D.D.A.) in collaboration with the Centers for Disease Control and Prevention, Atlanta, Georgia, USA.

The G and P types were determined by using reverse transcription–PCR (*5*) Samples that could not be typed were subjected to nucleotide sequencing. Amplification of the nonstructural protein NSP4 gene was conducted as described (*6*).

Rotavirus genomic RNAs were visualized by polyacrylamide gel electrophoresis (*7*) and staining with a Silver Stain II kit (Wako Pure Chemical Industries Ltd., Osaka, Japan) with a few modifications. The gel was incubated in staining solution for 45 min instead of 15 min, and in each washing step, the temperature of deionized water was maintained at 25°C.

VP7, the VP8* portion of outer capsid VP4, and NSP4 genes were sequenced by using the method previously described (*5*). Sequence similarity was searched with BLAST (www.ncbi.nlm.nih.gov/blast), multiple sequence alignment was conducted with ClustalW (www.ebi.ac.uk/clustalw), and phylogenetic trees were constructed by the neighbor-joining method using MEGA version 3.1 (*8*). Bootstrap analysis of 1,000 replicates was used to investigate branching of constructed trees. The *N*-glycosylation site of VP7 was predicted by using a NetNGlyc 1.0 Server (www.cbs.dtu.dk/services/NetNGlyc).

A total of 38 samples were obtained (31 from Khanh Hoa General Hospital and 7 from Ninh Hoa Hospital). G and P types were determined for 35 samples. G type could not be determined for 3 samples (KH142, KH228, and KH210). However, these samples were typed as G2, G4, and G5, respectively, by a homology search of VP7 gene sequence. Thirty-three samples were typed as G1P[8], 2 as G2P[4], 1 as G4P[6], 1 as G5P[6], and 1 as G9P[8]. Three different electropherotypes with a long pattern were identified in 20 electrophoretic-positive samples. Of these, 18 samples showed the same electropherotype and all were typed as G1P[8]. Each of the G4P[6] and G5P[6] rotaviruses showed different electropherotypes. The sample that contained G5 rotavirus was isolated from a 7-month-old girl admitted to Khanh Hoa General Hospital in 2004 with diarrhea, fever, and malnutrition.

Sequence analyses of the VP7 gene showed that KH210 had more than 80% nucleotide and 90% amino acid identities with representative G5 rotaviruses from humans and pigs (Table 1). When we compared VP7 antigenic regions A (aa 87–101), B (aa 143–152), C (aa 208–223), and F (aa 235–242) (*9*) with representative strains of human G5 rotaviruses, unique substitutions were observed at aa positions 217 (V) of region C and 235 (Y) in region F. Other unique substitutions observed in KH210 were C, I, S, and N at positions 6, 29, 112, and 119, respectively.

**Table 1:** Percentage identity of amino acid and nucleotide sequences of VP7 genes of KH210 and selected rotavirus G5 strains*

Strain	Species (country)	Deduced amino acid identity, %	Nucleotide identity, %
MRC 3105†	Human (Cameroon)	92	89
IAL-28	Human (Brazil)	91	85
CC117	Porcine (Argentina)	93	86
C134	Porcine (Argentina)	93	86
TFR-41	Porcine (Australia)	93	NA‡
JL94	Porcine (People’s Republic of China)	91	85
OSU	Porcine (USA)	91	84
A34	Porcine (Venezuela)	92	84
A46	Porcine (Venezuela)	92	82
134/04–15	Porcine (Italy)	91	83
H1	Equine (USA)	91	85

*GenBank accession nos.: MRC3105/2000, AY327107; IAL-28, L79916; CC117, L35056; C134, L35058; TFR-41, P32547; JL94, AY538665; OSU, X04613; A34, L35059; A46, L35054; 134/04–15, DQ062572; H1, AF242393. †Partial sequence. ‡NA, not available.

**Figure 1:**
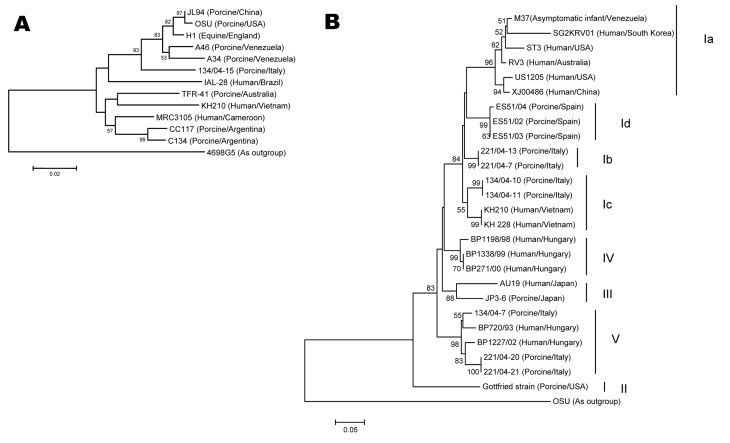
A) Phylogenetic tree constructed from deduced amino acid sequences of the VP7 gene of animal and human G5 rotaviruses. Strain 4695G5, an equine G3 strain, was used as an outgroup. Bootstrap values are expressed as percentages. Bootstrap value <50 is not shown. Strain KH210 clustered with the human G5 rotavirus from Cameroon (MRC3105) and other G5 rotaviruses of porcine origin from Australia and Argentina. The Brazilian human isolate of G5 rotavirus clustered with G5 rotaviruses of porcine and equine origin. Species of origin followed by country of isolation is shown in parentheses after the strain name. B) Phylogenetic tree constructed from the deduced amino acid sequences of the VP8* gene of rotaviruses representing all P[6] lineages. Strain OSU was used as an outgroup. Bootstrap values are expressed as percentages. A bootstrap value <50 is not shown. Strains KH210 and KH228 clustered with lineage Ic. Species of origin followed by country of isolation is shown in parentheses after the strain name. Scale bar shows genetic distance expressed as amino acid substitutions per site.

Phylogenetic analysis of the amino acid sequence of the VP7 gene (Figure 1A) of G5 strains showed 2 genetically distinct groups; KH210 clustered with a porcine G5 strain from Australia. This group also contained a human G5 strain from Cameroon. The human G5 strain from Brazil may belong to the other group. Similar to other G5 strains, VP7 of KH210 showed 1 potential *N*-glycosylation site at position 69.

The nucleotide and amino acid sequences of VP8* of strains KH210 and KH228 showed high identity (89%–93% and 90%–95%) with lineage I and relatively low identity (81%–86% and 85%–90%) with other lineages of P[6] (Table 2). Phylogenetic analysis of the amino acid sequence of VP8* of KH210, KH228, and other P[6] strains showed that Vietnamese strains clustered with Italian porcine strains of lineage Ic (Figure 1B).

**Table 2:** Percentage identity of the partial amino acid and nucleotide sequences of VP8* genes of KH210 and selected rotavirus P[6] strains*

Strain (lineage)	Species (country)	Deduced amino acid identity, %	Nucleotide identity, %
M37 (Ia)	Human (Venezuela)	90	89
221/04–7 (Ib)	Porcine (Italy)	94	92
134/04–10 (Ic)	Porcine (Italy)	95	93
ES51/04 (Id)	Porcine (Spain)	92	91
Gottfried (II)	Porcine (USA)	85	82
AU19 (III)	Human (Japan)	87	81
BP1198/98 (IV)	Human (Hungary)	90	86
BP1227/02 (V)	Human (Hungary)	88	85

*The amino acid and nucleotide sequence identity of VP8* genes of KH210 and KH228 were 99%. The amino acid sequence identity of VP8* genes between KH228 and Gottfried was 84%. Otherwise, the identity of amino acid and nucleotide sequences of VP8* genes of KH228 and selected rotavirus P[6] strains were same as for KH210. GenBank accession nos.: M37, L20877; 221/04–7, AY955303; 134/04–10, AY955299; ES51/04, AY955306; Gottfried, M33516; AU19, AB017917; BP1198/98, AJ621504; BP1227/02, AJ621505.

**Figure 2:**
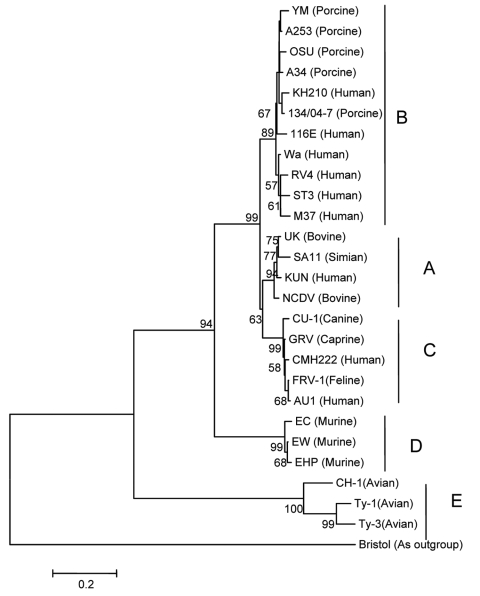
Phylogenetic tree constructed from the deduced amino acid sequences of the NSP4 gene of rotaviruses representing all genotypes. Strain Bristol, a group C rotavirus, was used as an outgroup. Bootstrap values are expressed as percentages. Bootstrap value <50 is not shown. Strain KH210 clustered with strains in genotype B. Species of origin is shown in parentheses after the strain name. Scale bar shows genetic distance expressed as amino acid substitutions per site.

Phylogenetic analysis of the amino acid sequence of NSP4 showed that KH210 was closely related to porcine rotaviruses and belonged to genotype B (Figure 2), with identity of 94% to 96% to porcine stains and 91% to 95% to human strains of genotype B. Similar to the structure of most rotaviruses, 2 potential *N*-glycosylation sites were located at aa positions 8 and 18 (*8*).

## Conclusions

G5 rotaviruses are isolated mainly from pigs. However, in 1994 these viruses were reported in samples from Brazilian children with diarrhea (*10*). Subsequently, G5 was identified as a cause of human infection in several states in Brazil (*11*), which suggests a broader distribution of this unusual serotype. Human G5 rotaviruses were then identified in children with acute diarrhea in Argentina (*12*) and Paraguay (*13*). Recently, human G5 rotavirus was reported in Cameroon (*4*), the first human G5 rotavirus isolated in Africa.

The human G5 rotavirus from Cameroon has a short RNA pattern. In contrast, the human G5 rotaviruses detected in Vietnam and Brazil have long RNA patterns. However, their VP7 genes are not in the same cluster in the phylogenetic tree. Brazilian G5 rotaviruses isolated from humans are usually found in combination with the P[8] genotype (*10*). This combination is a result of naturally occurring reassortment between human (P[8], Wa-like) and animal (G5, OSU-like) strains (*14*).

We have identified the combination of KH210 with genotype P[6]. This genotype is found predominantly in strains of porcine origin. VP8* of Vietnamese strains clustered with porcine strains isolated in Italy and belonged to the Ic lineage of P[6]. The NSP4 gene of KH210 belonged to genotype B and had high amino acid identities with both human and porcine rotaviruses of the same genotype. These findings are consistent with those of RNA-RNA hybridization (*7*) and sequence analysis (*15*). Human strain Wa and porcine strain OSU were shown to form 3 hybrid bands, including the one that corresponded to the NSP4 gene (*7*).

Rotaviruses from different species were shown by phylogenetic analysis to have similar NSP4 sequences that were clustered into the same NSP4 genotype. However, identifying the origin of the NSP4 gene in KH210 was not possible. On the basis of unique amino acid substitutions in VP7 and phylogenetic analysis of VP7 and VP8*, we believe that Vietnamese G5 strain most likely originated from porcine rotavirus by genetic reassortment or as whole virions. Although 2 strains with different G genotypes (G5 for KH210 and G4 for KH228) have identical VP8* genes (Figure 1B), which suggests a contemporary genetic reassortment event, it is not known whether sequence divergence between KH210 and porcine rotaviruses indicates interspecies transmission.

More information will be obtained from sequencing of contemporary porcine rotavirus strains in Vietnam. Identification of a human G5P[6] strain in Vietnam will provide a rationale for expanded surveillance to understand its prevalence in Vietnam and other Asian countries.
